# Planetary health training needs in Italy: an exploratory institutional survey within a national environment and health project

**DOI:** 10.3389/fpubh.2026.1866999

**Published:** 2026-09-30

**Authors:** Fabio Panariello, Sofia Mao, Tindara Scirocco, Mattia Di Russo, Giulia Congedo, Stefania Bruno, Chiara Cadeddu, Paola Angelini, Paolo Giorgi Rossi, Walter Ricciardi, Leonardo Villani

**Affiliations:** 1 Section of Hygiene, University Department of Life Sciences and Public Health, Università Cattolica del Sacro Cuore, Rome, Italy; 2 Erasmus School of Health Policy and Management, Erasmus University Rotterdam, Rotterdam, Netherlands; 3 Regional Health Authority of Emilia-Romagna, Bologna, Italy; 4 Epidemiology Unit, Azienda USL—IRCCS di Reggio Emilia, Reggio Emilia, Italy; 5 UniCamillus - Saint Camillus International University of Health and Medical Sciences, Rome, Italy

**Keywords:** education, environmental health, Italy, planetary health, public health, training needs

## Abstract

**Introduction:**

The Planetary Health approach, which recognizes the interdependence between human health and the integrity of natural systems, is increasingly considered essential for addressing health challenges related to climate change and environmental degradation. Integrating this perspective into the education and professional development of environment and health practitioners is a priority, especially in countries facing significant climate-related pressures. This study explores perceived Planetary Health training needs among Italian institutions working in the transdisciplinary field of environment and health.

**Methods:**

An exploratory survey was distributed among 21 partner institutions of a national Ministry of Health project on outdoor air quality and health, including universities, national research institutes, local health authorities, public health authorities, and regional environmental protection agencies. The questionnaire assessed perceived training needs, preferred training delivery formats, and target professional profiles. Descriptive statistics were used to analyse responses.

**Results:**

Fourteen institutions completed the survey (66.7%), including institutions from public health, environmental protection, research, and academic settings. The most frequently identified needs were understanding the health impacts of pollution (78.6%), stakeholder and public engagement in decision-making processes (64.3%), and advanced environmental epidemiology (57.1%). Preferred delivery formats included seminars (78.6%), short, practice-oriented specialized training courses (71.4%), and distance learning (50%). The range of professional profiles identified as potential participants suggests that future training initiatives may need to accommodate diverse professional backgrounds.

**Conclusion:**

This exploratory survey provides preliminary, project-based information that may inform the development of a postgraduate training course for professionals working within participating Italian environment and health institutions. The findings may guide the initial design of educational activities that combine structured and flexible learning options for the participating institutions.

## Introduction

Climate change and environmental degradation are increasingly recognized as major determinants of population health, contributing to a wide range of health outcomes, including non-communicable and infectious diseases (1, 2). These impacts disproportionately affect vulnerable populations and represent a significant health burden (3, 4). Addressing these complex and interconnected challenges requires integrated approaches that bridge environmental and health domains, combining scientific evidence with institutional coordination and adequately trained professionals (5).

In this context, Planetary Health has emerged as a transdisciplinary framework aimed at understanding and addressing the interconnections between human health and the integrity of natural systems (1). Beyond its conceptual foundations, the Planetary Health approach calls for systems thinking, intersectoral collaboration, and solution-oriented strategies, highlighting the need for competencies that enable professionals to translate environmental and health evidence into prevention and policy-making processes (6). Consequently, strengthening education and training has become a central priority within the Planetary Health agenda (6).

Internationally, Planetary Health education courses have been increasingly emerging within academic curricula and professional development pathways (7, 8). In Italy, however, structured postgraduate and professional training programs in this area remain limited, although emerging initiatives exist (9, 10). This gap is particularly relevant given Italy’s exposure to climate-related health risks: in 2024, the Italian population experienced an average of 46 heatwave days, while 60.9% of the national land area experienced at least 1 month of extreme drought annually during 2020–2024 (11); moreover, persistent air-quality challenges remain particularly pronounced in the Po Valley, where geographic and meteorological conditions favor pollutant accumulation (12). In parallel, the transmission of vector-borne diseases is expanding, reflecting the broader climate-driven expansion of vector habitats across Europe (13).

In response to these challenges, Italy has strengthened its institutional health and environment framework through the consolidation of the integrated network comprising the National Health Prevention System from Environmental and Climate Risks (SNPS) and the National System for Environmental Protection (SNPA). This network plays a strategic role in integrating environmental monitoring, risk assessment, and public health prevention activities, and is also involved in several national initiatives aimed at reinforcing research and professional capacity in the fields of environment, climate, and health (14, 15). Within this evolving architecture, education and professional training are identified as essential components for strengthening competencies and supporting the implementation of integrated environment and health policies (14).

Among the research initiatives, the project “Aria outdoor e salute: un atlante integrato a supporto delle decisioni e della ricerca*”* (“Outdoor Air and Health: An Integrated Atlas to Support Decision-Making and Research”) aims to develop an integrated digital tool combining air quality indicators with health outcomes, and to support evidence-informed policymaking through advanced data tools (16). Importantly, a core task involves several educational activities, including the development of a postgraduate advanced training course for professionals working within institutions involved in environmental protection, public health, and related policy processes (16). In this context, an exploratory survey was conducted among Italian project institutions to inform course design by assessing training needs, preferred formats, and professional profiles.

This study presents those findings as a preliminary step toward developing an effective, context-sensitive, interdisciplinary postgraduate course.

## Materials and methods

An online exploratory survey was conducted within the framework of the Italian National Project “Aria outdoor e salute: un atlante integrato a supporto delle decisioni e della ricerca”. The survey targeted Italian institutions involved in the project, including universities, national research institutes, local health authorities, public health authorities, and regional environmental protection agencies.

### Survey design

The questionnaire was developed to support a project-based assessment of institutional training activities and perceived training needs in the field of environment and health, with the specific aim of informing the design of a postgraduate advanced training course within the “Aria outdoor e salute: un atlante integrato a supporto delle decisioni e della ricerca” project. The development process followed a four-stage, evidence-informed and expert-reviewed approach.

In the first stage, two researchers with expertise in environmental health, Planetary Health, and public health education prepared an initial item pool and draft questionnaire. The development of the questionnaire was informed by an exploratory review of relevant Planetary Health education literature and frameworks, including the Planetary Health Education Framework, as well as publicly available institutional resources and educational materials (6, 17). This review helped identify recurring thematic areas, relevant professional audiences, and commonly used training delivery formats (17).

In the second stage, the draft questionnaire was circulated to four senior members of the project team with expertise in environmental health, public health, and professional education. Reviewers assessed the proposed items with regard to relevance to the project’s educational objectives, clarity of wording, completeness of the thematic coverage, appropriateness of response options, and practical feasibility for institutional respondents. Feedback was collected through written comments and group discussion.

In the third stage, the two researchers revised the questionnaire by incorporating the feedback received. Revisions included rewording selected questions, refining response options, and reorganizing items to improve clarity and alignment with the planned course-development process. The revised version was subsequently resubmitted to the same experts for a further round of review.

In the final stage, the questionnaire was approved by all four reviewers before distribution to participating institutions. The resulting instrument was designed as a pragmatic, project-specific tool to explore institution-level training activities and perceived training needs. It was not intended as a psychometrically validated measurement scale; rather, it served as an exploratory needs-assessment tool to support the planning of the project’s educational activities.

### Structure of the survey

The survey consisted of 17 questions organized into five thematic sections: institutional profile; training activities conducted by the institution; institutional training needs; training courses delivered or planned within PNC Projects; and institutional contribution to the “Atlante Aria e Salute” advanced training course. Questions were predominantly closed-ended and multiple-choice. A limited number of open-ended questions were also included.

The first section collected institutional information, including the type of institution, its role within the Project, and the professional backgrounds of staff involved in the institutions’ activities. The second section investigated whether the institution organizes training activities related to environment and health, the target audiences of such activities, the delivery formats, and the availability of institutional platforms for training promotion. The third section focused on perceived training needs, exploring the types of training considered most necessary for the development of a structured course, as well as the professional profiles within the institution identified as potential recipients of training activities. The fourth section assessed institutional participation in training activities within other national projects. Finally, the fifth section explored the institution’s interest in contributing to future educational initiatives, including the potential contribution to a postgraduate training course and the areas of expertise the institution could provide. The full survey questionnaire is provided in the Supplementary File S1.

### Survey participants and administration

A total of 21 institutions participating in the “Aria outdoor e salute” project were invited to complete the survey. Each participating institution corresponded to one operational unit within the project. The questionnaire was distributed to the designated contact person of each operational unit, who personally completed the questionnaire and provided an institution-level response based on their knowledge of the institution’s training activities, perceived training needs, and potential contribution to the planned educational initiative. The unit of analysis was the institution rather than the individual respondent. The reported professional backgrounds of institutional staff included public health, environmental sciences, medical disciplines, and technical fields, such as engineering, statistics, and mathematics. The survey was delivered between August and September 2024, and the estimated completion time was approximately 10 min.

### Analysis of survey data

Data were analyzed using descriptive statistical methods in Microsoft Excel. Absolute frequencies and percentages were calculated to summarize institutional characteristics, perceived training needs, target professional profiles, and preferred training formats. Responses to open-ended questions were manually grouped into categories based on shared content.

### Ethics approval and consent to participate

The survey was developed and underwent iterative expert review as part of the “Aria outdoor e salute: un atlante integrato a supporto delle decisioni e della ricerca” project. Its distribution to participating institutions was approved and authorized by the Italian Ministry of Health as an integral part of the project activities. All participants took part voluntarily and free of charge, giving their consent to complete the survey.

## Results

### Characteristics of responding institutions

The responding institutions included five institutional categories (Table 1). Regional Public Health and Prevention Authorities represented the largest group (6/14, 42.9%), followed by Local Health Authorities (3/14, 21.4%), while Universities and Regional Environmental Protection Agencies each accounted for 2/14 institutions (14.3%); one National Research Institute also participated (7.1%). The multidisciplinary nature of the responding institutions is reflected in the professional backgrounds reported (Supplementary Table S1 in Supplementary material), which spanned five broad domains: clinical and public health professions, epidemiological and quantitative sciences, laboratory and biomedical sciences, environmental and technical professions, and academic, policy and communication roles. Reported professional profiles ranged from public health medical doctors, statisticians, and biologists – the most frequently reported (64.3, 57.1 and 50% of institutions respectively) – to less common but specialized profiles such as chemists, economists, and communication professionals (7.1–21.4%).

**Table 1:** Classification of responding institutions by institutional type (*N* = 14).

Type of institutions	*n*	%
Regional Public Health and Prevention Authorities	6	42.9%
Local Health Authorities	3	21.4%
Regional Environmental Protection Agencies	2	14.3%
Universities	2	14.3%
National Research Institutes	1	7.1%

### Training needs in planetary health

Figure 1 illustrates the perceived training needs identified by responding institutions in relation to the development of a postgraduate advanced training course in Planetary Health.

**Figure 1:**
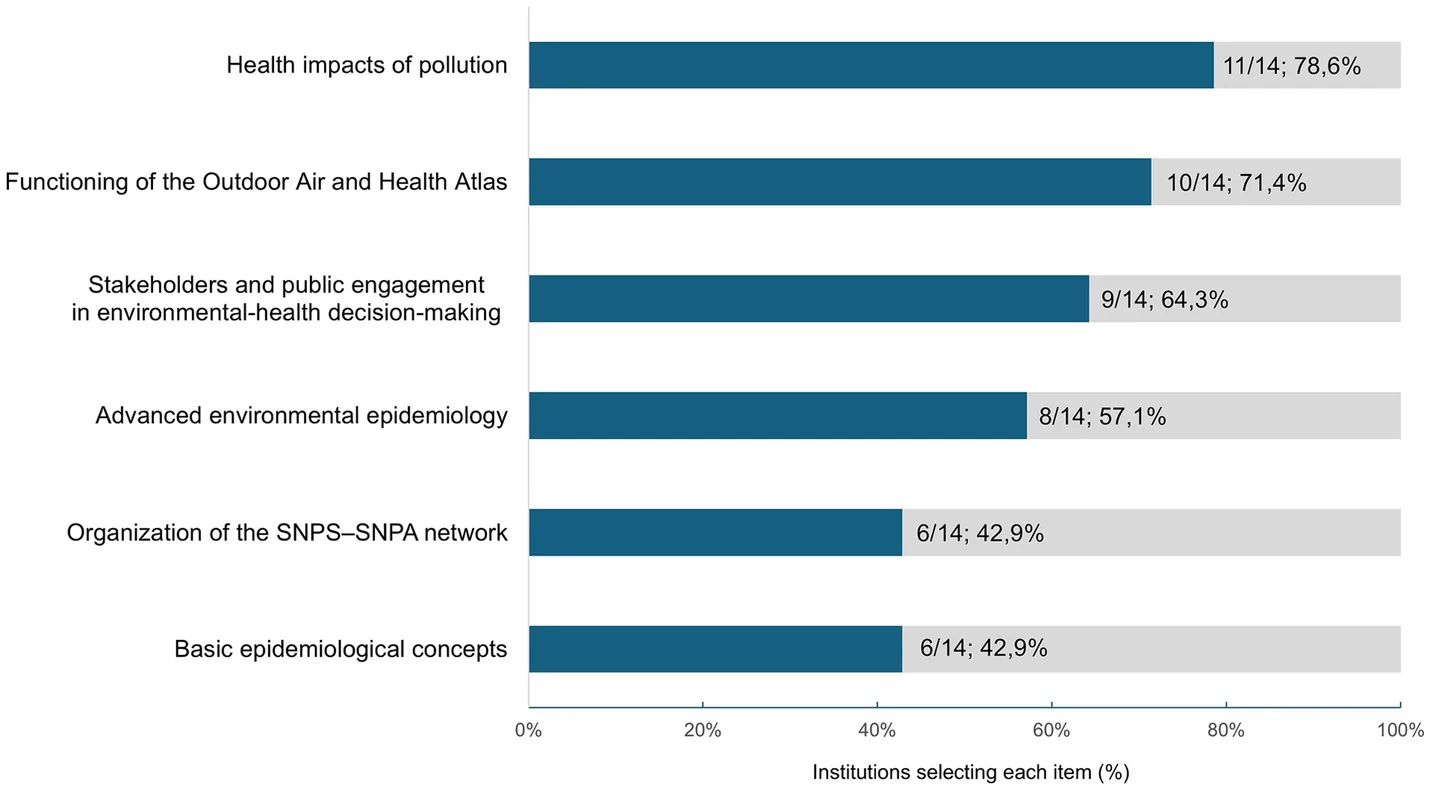
Institution-reported priority training areas for the planned postgraduate course (*N* = 14). Each institution could select more than one training area. Bars show the proportion and number of responding institutions that selected each area; therefore, percentages do not sum to 100%. The stakeholder and public engagement item refers to training in methods for involving institutional stakeholders and members of the public in environmental-health decision-making. Training areas selected by one institution only are not shown.

The most frequently reported need was training on the health impacts of pollution (*n* = 11, 78.6%). Training in methods for stakeholder and public engagement in environmental-health decision-making was identified as a priority by nine institutions (64.3%). Advanced environmental epidemiology was considered a priority training need by eight institutions (57.1%), whereas six institutions (42.9%) highlighted the need for training on basic epidemiological concepts. Six institutions also reported the need to learn more about the organization of the SNPS-SNPA network. Other training needs included legal and economic aspects of environmental health, toxicology of major environmental pollutants, and health impact assessment, each reported by one institution (7.1%). Finally, 10 institutions (71.4%) selected the inclusion of the “Outdoor Air and Health Atlas” as a potential component of the planned training course.

Individual professional profiles identified by responding institutions as potential participants in the planned postgraduate training course are reported in Figure 2. Multiple selections were allowed.

**Figure 2:**
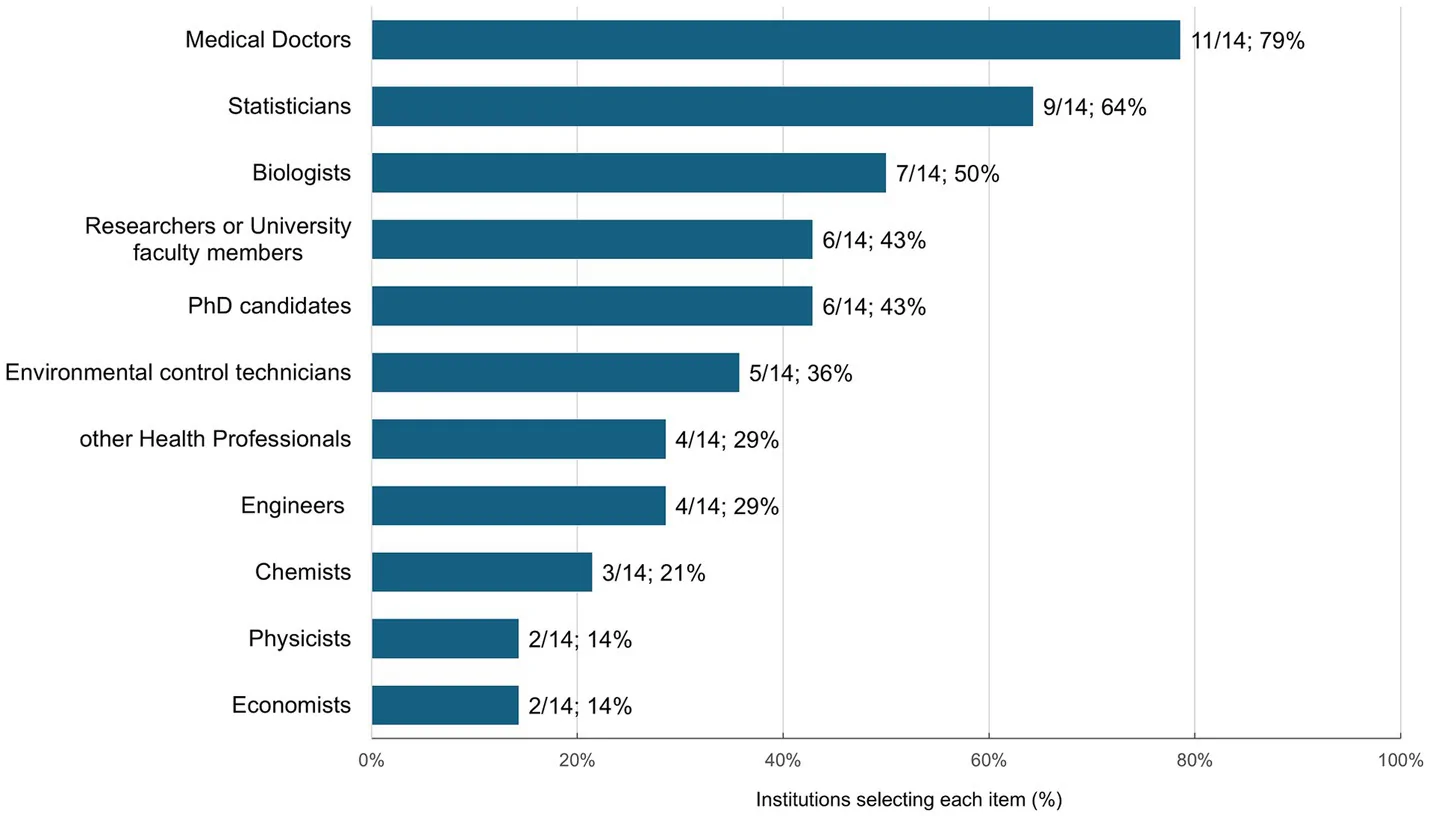
Individual professional profiles identified by responding institutions as potential participants in the planned postgraduate training course (*N* = 14). Each institution could select more than one item. Bars show the proportion and number of responding institutions that selected each item; therefore, percentages do not sum to 100%. Professional profiles selected by one institution only are not shown.

Medical doctors were identified as potential participants by 11 institutions (78.6%), followed by statisticians (9/14, 64.3%) and biologists (7/14, 50.0%). Researchers or university faculty members and PhD candidates were each identified by six institutions (42.9%). Other reported profiles included health professionals, environmental control technicians, engineers, mathematicians, physicists, chemists, economists, communication professionals, and professionals working in environmental and occupational prevention. In addition to individual professional profiles, some institutions also identified universities, schools, and civil society as potential broader audiences for educational or engagement activities. As these categories do not represent individual professional profiles, they were not included in Figure 2.

Figure 3 illustrates the preferred training formats expressed by the responding institutions. Seminars emerged as the most frequently requested format, selected by 11 institutions (78.6%), followed by short, practice-oriented specialized training courses (10 institutions, 71.4%). Half of the institutions indicated a preference for distance learning formats. Master’s degree programs and postgraduate advanced training programs were each selected by four institutions (28.6%), while PhD programs were considered a relevant training format by two institutions (14.3%). Finally, one institution (7.1%) expressed interest in receiving informational materials specifically designed for healthcare organizations.

**Figure 3:**
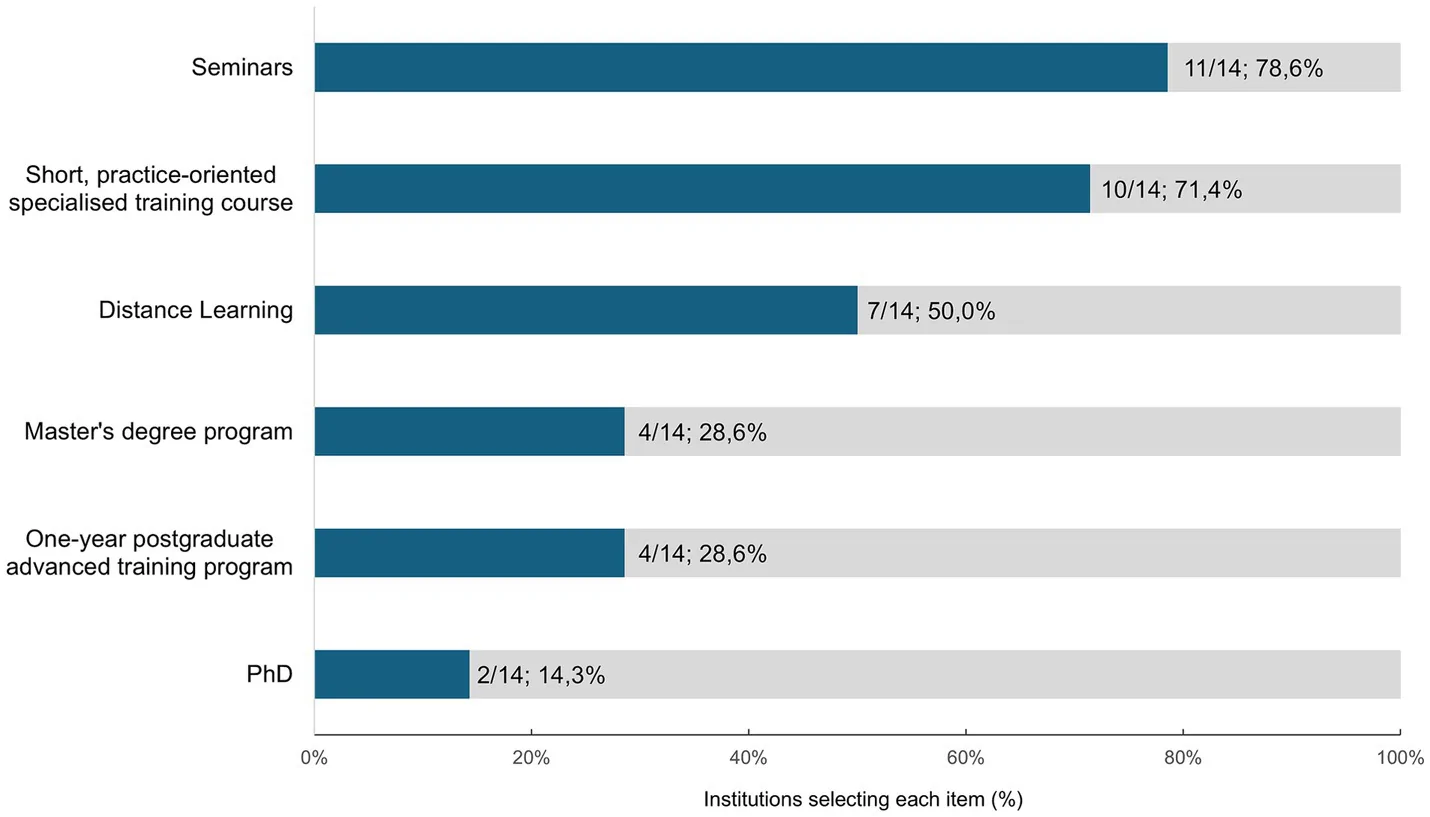
Preferred training formats for Planetary Health education among responding institutions (*N* = 14). Institutions could select more than one training format. Bars represent the percentage of institutions that expressed a preference for each format.

### Institutional experience in environment and health training and expertise available for course development

Most participating institutions reported experience in training activities related to environment and health. Specifically, 11 out of 14 institutions (78.6%) identified the presence of ongoing or previous educational activities in this field, reporting professional groups they already targeted with such initiatives. In addition, eight institutions provided information on areas of expertise they would be willing to contribute to the development of a postgraduate advanced training course. These competencies, collected through open-ended responses, were grouped into four main thematic areas. The epidemiological and methodological area included expertise in general and environmental epidemiology, GIS systems, research methodologies, and both basic and advanced data analysis methods. The organizational and governance area focused on the structure and functioning of the SNPS-SNPA network, as well as coordination mechanisms between research projects and national funding programs. The technical and environmental area comprised competencies related to air pollution, including emission sources, pollutants, temporal trends, and environmental monitoring activities. Finally, the public health and communication area covered integration with Regional Prevention Plans and the dissemination of knowledge at the local level.

## Discussion

This study offers insights into the perceived training needs in environmental health and Planetary Health as identified by 14 Italian institutions involved in a national project.

The responding institutions included several institutional categories and reported a range of professional backgrounds. However, Regional Public Health and Prevention Authorities constituted the largest group, and all participating institutions were involved in the same national project; the findings should therefore be interpreted as reflecting the perspectives of this purposively selected institutional network.

Planetary Health education requires collaboration across disciplinary and professional boundaries to address the interconnected environmental, social, and health dimensions of contemporary challenges (18). Against this broader background, the findings of this exploratory survey provide preliminary information that may inform the design of context-specific educational activities for the participating institutions.

Notably, pollution and its health impacts emerged as the most prominent training need identified by responding institutions, consistent with the persistent relevance of air and water pollution in the Italian context (11, 12).

A substantial proportion of institutions emphasized the importance of epidemiology training, both at the basic level (43%) and advanced environmental level (57%), highlighting the central role of epidemiological competencies within the Planetary Health framework. Environmental epidemiology encompasses the study of exposure–outcome relationships between environmental and climate-related hazards (e.g., air and water pollutants, extreme temperatures, vector-borne pathogens) and human health, integrating methods from exposure assessment, biostatistics, and risk analysis. Strengthening epidemiological training in this context is essential to support the integrated analysis of environmental and health data, the assessment of complex and cumulative risks, and the generation of evidence capable of informing prevention strategies and policy decisions (19, 20). In this perspective, closer integration between epidemiology, climate science, and environmental health within postgraduate and professional training pathways appears particularly relevant (10, 21).

Furthermore, the need for training in stakeholder engagement and public participation underscores the collaborative nature of Planetary Health. In this context, professionals are required to develop skills that support effective communication and collaboration with a wide range of stakeholders, including policymakers, community organizations, researchers, and professionals from sectors such as urban planning and agriculture (22). Strengthening competencies in stakeholder engagement is essential to promote cross-sectoral collaboration and to facilitate the translation of scientific evidence into policy-relevant and actionable interventions (22–24).

The identified need for training related to the Italian environmental and health governance context (SNPS-SNPA network) further underscores the importance of situating Planetary Health education within national institutional frameworks. A comprehensive and clearer understanding of the roles, responsibilities, and interactions of the institutions involved is crucial for the effective design and implementation of environmental health policies and interventions (25).

Finally, the ability to harness large-scale environmental and health datasets is becoming increasingly critical for tracking trends, assessing risks, and informing decision-making processes among professionals attending postgraduate and advanced training programs (26, 27).

Importantly, these training needs emerge in a context where many participating institutions reported prior experience in organizing education and training activities related to environment and health. Within this project-based institutional network, the findings suggest perceived interest in further structuring and integrating existing educational initiatives within a Planetary Health-oriented framework.

Concerning preferred learning formats and target audiences for training initiatives, the reported preferences suggest that a future course designed for the participating institutions may benefit from combining shorter, practice-oriented and flexible learning options.

Responding institutions expressed a clear preference for seminars and short, practice-oriented specialized training courses. Seminars can facilitate expert engagement, real-time discussion, and knowledge exchange on emerging Planetary Health topics, while short, practice-oriented specialized training courses offer opportunities for skill development, specialization, and career advancement (7, 28, 29). At the same time, the interest expressed in online and distance learning, selected by half of the institutions, underscores the importance of accessibility and flexibility, which are essential features for professionals already engaged in institutional and operational roles (28). The coexistence of different preferred formats may reflect the range of professional profiles identified by the participating institutions, including health professionals, technical experts, researchers, and institutional staff. Overall, these results suggest that effective Planetary Health capacity-building initiatives should combine structured training programs with more modular and accessible learning opportunities, in line with existing evidence supporting experiential, participatory, and context-sensitive approaches to interdisciplinary education (5).

The training needs identified in this study are consistent with broader European efforts aimed at strengthening education, research, and capacity building in Planetary Health. Over the past decade, several initiatives have emerged worldwide to promote interdisciplinary approaches, integrate environmental and health knowledge, and support the translation of scientific evidence into policy and practice, such as the Planetary Health Alliance and the Lancet Countdown on Health and Climate Change (30, 31). In parallel, several European countries have begun to institutionalize Planetary Health within higher education and professional training, including postgraduate programs in public and global health as well as the progressive integration of climate and environmental health topics into undergraduate medical curricula (8, 28, 32). Within this evolving context, these preliminary findings may inform the development of context-specific educational activities for professionals working at the interface of environmental protection and public health.

### Study limitations

This study has several limitations that should be considered when interpreting the findings. First, the relatively small sample size, comprising 14 institutions, limits the generalizability of the results to the broader Italian context. The institutions were purposively selected as partners in a national project focused on the environment and health and may therefore differ from institutions not involved in this project. Accordingly, the findings should be interpreted as reflecting the perspectives of this project-based institutional network rather than those of the wider Italian institutional landscape.

Second, each questionnaire was completed by a single designated contact person for the participating operational unit, corresponding to one institution. Although respondents were asked to provide an institution-level perspective, a single informant may not fully capture the diversity of training activities, priorities, and perspectives within an institution. Individual role, knowledge of institutional activities, and professional background may also have influenced the responses. As respondent-level characteristics were not systematically collected and responses were not independently verified within institutions, the extent of potential reporting bias could not be assessed.

Third, the questionnaire was developed as a pragmatic, project-specific needs-assessment tool and was not intended as a psychometrically validated measurement scale. Although its development was informed by relevant Planetary Health education literature and frameworks and underwent iterative expert review, its reliability, construct validity, and other measurement properties were not formally assessed.

Finally, the endorsement of the “Outdoor Air and Health Atlas” as a component of the planned training course, reported by 10 of 14 institutions (71.4%), should be interpreted cautiously. As the participating institutions were involved in the development and implementation of the Atlas within the project, this finding may reflect familiarity with and institutional commitment to the tool rather than an independent assessment of its educational value or training relevance.

Future research could complement these findings by incorporating more granular assessments of individual competencies, objective evaluations of skill gaps, or mixed-method approaches combining quantitative and qualitative data. Despite these limitations, this exploratory survey provided a valuable evidence base to inform the development of training and educational initiatives within the framework of the “Aria Outdoor e Salute” project and other activities related to environment, climate, and health. In this sense, the study offers a pragmatic and context-sensitive contribution to the emerging field of Planetary Health education in Italy.

## Conclusion

This survey provided preliminary but actionable insights into Planetary Health training needs among Italian institutions. Participating institutions identified specific training priorities that integrate epidemiological, environmental, and system-level competencies.

Our findings may inform the initial design of a context-sensitive postgraduate training course for professionals working within the participating institutions. This survey did not assess inter-institutional collaboration. However, Planetary Health may offer a useful conceptual framework for future collaborative educational initiatives across organizations working at the interface of environmental protection and public health in Italy.

## Data Availability

The raw data supporting the conclusions of this article will be made available by the authors, without undue reservation.
