# Supplementary material for: Planetary health training needs in Italy: an exploratory institutional survey within a national environment and health project
Source: Front Public Health. 2026 Sep 30;14:1866999. doi: 10.3389/fpubh.2026.1866999 (PMC13661544; doi:10.3389/fpubh.2026.1866999)
Supplement: Supplementary file 1 [file Table_1.docx]

**Supplementary Materials**

**Supplementary File S1. Survey – Planetary Health Training Needs Assessment**

**Translated version**

**Outdoor Air and Health Atlas: Institutional Profile Form for the Activation of an Advanced Training Course**

**Section 1. Institutional Profile**

*This section is intended to provide a brief description of the respondent organization or institution, its representatives, and its role within the project.*

**I. Name of Institution:** _________________________________

**II. Contact Person:** __________________________________

**III. Role in the definition of Work Package 6 “Professional Training”:** ___________________________________________

**IV. Contact Person for WP6 at the Institution:** ____________________________________________________________

**V. Professional profiles involved in the project or engaged in the use or production of 'environment and health' data in your Institution:**

- MD specialized in Hygiene and Preventive Medicine and Public Health
- MD specialized in Respiratory Medicine
- MD specialized in Clinical Pathology and Clinical Biochemistry
- MD specialized in Clinical Pharmacology and Toxicology
- MD specialized in Microbiology and Virology
- MD specialized in Anatomical Pathology
- MD specialized in Health Statistics and Biometry
- MD specialized in Medical Physics
- MD specialized in Occupational Medicine
- MD specialized in Forensic Medicine
- Veterinary Medicine Professionals
- Health professionals specialized in Healthcare Assistance
- Health professionals specialized in Nursing Sciences
- Health professionals specialized in Environmental and Occupational Prevention Techniques
- Biologists
- Chemists
- Pharmacists
- Physicists
- Mathematicians
- Statisticians
- Economists
- Engineers
- Environmental Control Technicians
- Laboratory Technicians
- Ecologists
- Researchers or University Faculty Members
- Communication Professionals
- Other: ________________________________________________

**VI. Role of the institution in the 'Outdoor Air and Health Atlas' project:**

- Data provider
- Modelling and development of the Atlas
- Participation in the steering committee
- Involvement of citizens or civil society
- Professional training and communication
- Other: _________________________________________

**Section 2. Training Activities Conducted by the Institution**

*This section provides detailed information on training initiatives conducted by the institution, highlighting its commitment to education, training, and professional development.*

**I. The institution organizes training activities related to 'environment and health' addressed to:**

- National Health Service (NHS) physicians
- General Practitioners
- Primary Care Paediatricians
- Health professionals
- Biologists
- Chemists
- Pharmacists
- Physicists
- Mathematicians
- Statisticians
- Economists
- Engineers
- Environmental Control Technicians
- Laboratory Technicians
- Ecologists
- Environmental Educators
- Researchers or University Faculty Members
- Communication Professionals
- Schools
- Teachers
- Universities
- Civil society (e.g. public engagement events)
- Other: ______________________________________

**II. Courses are delivered through:**

- Master’s degree programmes
- Advanced training courses
- Seminars
- Distance learning courses (FAD)
- Webinars
- Meetings
- Lectures
- Other: ______________________________________

**III. Title and web link (if available) of relevant events, courses, and training activities delivered in the past year:** ______________________________

**IV. Title of events, courses, and training activities planned within the PNC project:** _____________________________________________

**V. Does the institution have a platform for promoting professional training activities?**

- Yes
- No

**VI. Additional comments or notes:** __________________________________________________

**Section 3. Institutional Training Needs in the 'Environment and Health' Area**

*This section explores and outlines the specific Planetary Health training needs identified by the institution in the field of environment and health.*

**I. Which training delivery format does your institution consider most appropriate for strengthening competencies in the field of environment and health?**

- Distance learning (online courses)
- Seminars
- PhD programmes
- Short, practice-oriented specialised training course
- One-year postgraduate advanced training programme
- Master’s degree programme
- Other: ________________________________________

**II. Training needs considered necessary for the structuring of a Planetary Health post-graduated advanced course:**

- Basic epidemiological concepts
- Health impacts of pollution
- Organization of the SNPS–SNPA network
- Advanced environmental epidemiology
- Stakeholder and public engagement in decision-making processes
- Functioning of the Outdoor Air and Health Atlas
- Other: ____________________________

**III. Professional profiles identified as target audiences for the post-graduated training course, belonging to the institution:**

- Medical Doctors
- Health professionals
- Biologists
- Chemists
- Pharmacists
- Physicists
- Mathematicians
- Statisticians
- Economists
- Engineers
- Environmental Control Technicians
- Technical professionals
- Ecologists
- Environmental Educators
- Researchers or University Faculty Members
- PhD Candidates
- Communication Professionals
- Schools – Teachers and Students
- Universities – Professors and Students
- Civil society
- Other: ____________________________________________________________

**Section 4. Training Courses Delivered or Planned within Other PNC Projects in the 'Environment and Health' Area**

**I. Does the institution provide/will it provide training activities within other PNC projects?**

- Yes
- No

**II. What type of activities have been/will be carried out?** _____________________________________________

**Section 5. Institutional Contribution to the Advanced Training Course**

*This section investigates the potential contribution of each institution to the delivery of the advanced training course.*

I. Is the institution interested in contributing to the delivery of the Planetary Health advanced training course through teaching activities?

- Yes
- No

II. Briefly describe the expertise that the institution can provide for the delivery of the Planetary Health advanced training course (legal, economic, epidemiological fields, etc.):

______________________________________________________________________________________

**Italian version (original Language)**

**Survey – Identificazione dei Bisogni Formativi in ambito Planetary Health**

**Atlante Aria Outdoor e Salute**: **Scheda presentazione Ente in tema di formazione per l'attivazione di un corso di perfezionamento**

**Sezione 1. Presentazione Ente**

*La sezione è dedicata a fornire una breve descrizione dell'organizzazione o dell'ente in questione e i suoi responsabili e il suo ruolo nel progetto.*

**I. Nome Ente:** _________________________________

**II. Referente: __________________________________**

**III. Ruolo nella definizione dell’Obiettivo 6: ___________________________________________**

**IV. Referente Formazione: ____________________________________________________________**

**V. Figure professionali impiegate nel progetto oppure impiegate nell'uso o produzione dei dati “ambiente e salute”:**

- Medici con specializzazione in Igiene e Medicina Preventiva
- Medici con specializzazione in Malattie dell’Apparato Respiratorio
- Medici con specializzazione in Patologia Clinica e Biochimica Clinica
- Medici con specializzazione in Farmacologia e Tossicologia Clinica
- Medici con specializzazione in Microbiologia e Virologia
- Medici con specializzazione in Anatomia Patologica
- Medici con specializzazione in Statistica sanitaria e Biometria
- Medici con specializzazione in Fisica Medica
- Medici con specializzazione in Medicina del lavoro
- Medici con specializzazione in Medicina Legale
- Professionista in Medicina Veterinaria
- Professionisti sanitari specializzati in Assistenza sanitaria
- Professionisti sanitari specializzati in Scienze infermieristiche
- Professionisti sanitari specializzati in Tecniche della Prevenzione nell’Ambiente e nei luoghi di lavoro
- Biologi
- Chimici
- Farmacisti
- Fisici
- Matematici
- Statistici
- Economisti
- Ingegneri
- Tecnici del Controllo ambientale
- Tecnici di laboratorio
- Ecologi
- Ricercatori o Docenti universitari
- Professionisti in Comunicazione
- Altro: __________________________________________

**VI. Ruolo dell’ente nel progetto “Atlante Aria Outdoor e Salute”:**

- Fornitore di dati
- Modellizzazione e creazione dell’Atlante
- Partecipazione alla cabina di regia
- Coinvolgimento dei cittadini o società civile
- Formazione e comunicazione
- Altro: _________________________________________

**Sezione 2. Attività formative svolte dall’Ente**

*La sezione serve a fornire dettagli approfonditi sulle iniziative formative condotte dall'ente. Questa sezione è fondamentale per evidenziare l'impegno dell'organizzazione nella promozione dell'istruzione, della formazione e dello sviluppo professionale.*

**I. L’ente organizza attività formative inerenti “ambiente e salute” rivolte a:**

- Medici del SSN
- Medico di Medicina Generale
- Pediatra di Libera scelta
- Professionisti sanitari
- Biologi
- Chimici
- Farmacisti
- Fisici
- Matematici
- Statistici
- Economisti
- Ingegneri
- Tecnici del Controllo ambientale
- Tecnici di laboratorio
- Ecologi
- Educatori ambientali
- Ricercatori o Docenti universitari
- Professionisti in Comunicazione
- Scuole
- Docenti
- Università
- Società civile (ad esempio eventi divulgativi)
- Altro: ______________________________________

**II. I corsi sono fruibili attraverso:**

- Corsi di Master
- Corsi di perfezionamento
- Seminari
- Corsi FAD
- Webinar
- Incontri
- Lezioni
- Altro: ______________________________________

**III. Titolo e link alla pagina web (se presente) di eventi, corsi e attività formative realizzate dall'ente nell'ultimo anno che si ritengono rilevanti e di maggiore attinenza**: ______________________________

**IV. Titolo di eventi, corsi e attività formative che si intende realizzare nell'ambito del progetto PNC**: _____________________________________________

**V. L’Ente è dotato di una piattaforma per la promozione delle attività formative?**

- Si
- No

**VI. Aggiungere eventuali commenti o note: __________________________________________________**

**Sezione 3. Bisogni formativi dell’Ente in area “ambiente e salute”**

*La sezione è dedicata a esplorare e delineare i bisogni specifici di formazione che l'ente riconosce nell'ambito dell'ambiente e della salute. Questa sezione fornisce un'opportunità per comunicare i bisogni dell'organizzazione e le esigenze emergenti nel settore Planetary Health.*

**I. Quale modalità di erogazione formativa è maggiormente preferita dall’Ente per lo sviluppo di competenze nell’ambito “ambiente e salute”?**

- Formazione a distanza (FAD)
- Seminari
- Dottorato di ricerca
- Corso intensivo professionalizzante su tematiche specifiche
- Corso di perfezionamento annuale
- Master universitario
- Altro: ________________________________________

**II. Bisogni formativi percepiti come necessari nella strutturazione del corso di perfezionamento:**

- Concetti epidemiologici di base
- Nozioni di impatto dell’inquinamento sulla salute
- Organizzazione del SNPS-SNPA
- Epidemiologia ambientale avanzata
- Metodi per il coinvolgimento degli stakeholder e del pubblico nei processi decisionali
- Funzionamento dell’Atlante “Aria Outdoor e Salute”
- Altro: ____________________________

**III. Profili professionali identificati come target per il corso di perfezionamento, appartenenti all'ente:**

- Medici
- Professionisti sanitari
- Biologi
- Chimici
- Farmacisti
- Fisici
- Matematici
- Statistici
- Economisti
- Ingegneri
- Tecnici del Controllo ambientale
- Tecnici
- Ecologi
- Educatori ambientali
- Ricercatori o Docenti universitari
- Dottorandi
- Professionisti in Comunicazione
- Scuole – Docenti
- Università – Docenti Universitari
- Società civile
- Altro: ____________________________________________________________

**Sezione 4. Corsi di Formazione effettuati o in programma nell'ambito di altri PNC, in area "ambiente e salute"**

**I. L' Ente eroga/erogherà attività di formazione nel contesto di altri PNC?**

- si
- no

**II. Che tipo di attività sono state/saranno svolte? _____________________________________________**

**Sezione 5. Contributo dell'Ente al Corso di perfezionamento**

*La sezione investiga quale possa essere il contributo di ciascun Ente nell'erogazione di un corso di perfezionamento.*

**I. L'ente è interessato a contribuire all'erogazione del corso di perfezionamento attraverso le attività didattiche?**

- si
- no

**II. Descrivere brevemente le competenze che l'Ente mette a disposizione per l'erogazione del corso di perfezionamento (ambito legale, economico, epidemiologico, ecc)**

**______________________________________________________________________________________**

**Table S1.** Multidisciplinary professional backgrounds of the participating institutions.

| **Background** | **Professional title** | **n** | % |
| --- | --- | --- | --- |
| Clinical and Public Health Professionals | MD specialized in Hygiene and Preventive Medicine and Public Health | 9 | 64,3% |
|  | MD specialized in Occupational Medicine | 2 | 14,3% |
|  | MD specialized in Respiratory Medicine | 1 | 7,1% |
|  | Health professionals specialized in Nursing Sciences | 1 | 7,1% |
|  | Health professionals specialized in Healthcare Assistance | 1 | 7,1% |
|  | Veterinary Medicine Professionals | 1 | 7,1% |
|  | Psychologists | 1 | 7,1% |
| Epidemiological and Quantitative Sciences | Statisticians | 8 | 57,1% |
|  | Mathematicians | 3 | 21,4% |
|  | MD specialized in Health Statistics and Biometry | 2 | 14,3% |
|  | Epidemiologists | 1 | 7,1% |
| Laboratory and Biomedical Sciences | Biologists | 7 | 50,0% |
|  | Physicists | 2 | 14,3% |
|  | Chemists | 1 | 7,1% |
|  | Natural Scientists | 1 | 7,1% |
|  | MD specialized in Microbiology and Virology | 1 | 7,1% |
| Environmental and Technical Professions | Engineers | 3 | 21,4% |
|  | Health professionals specialized in Environmental and Occupational Prevention Techniques | 2 | 14,3% |
|  | Environmental Control Technicians | 1 | 7,1% |
|  | Architects | 1 | 7,1% |
| Academic, Policy and Communication Domain | Researchers or University Faculty Members | 4 | 28,6% |
|  | Communication Professionals | 3 | 21,4% |
|  | Economists | 2 | 14,3% |

Each institution could report more than one professional background, reflecting the interdisciplinary nature of the entities. Percentages indicate the proportion of institutions employing professionals with each background, not the overall distribution of professionals.
